# Lessons learned about virtual education during the COVID-19 pandemic*

**DOI:** 10.17533/udea.iee.v41n2e01

**Published:** 2023-08-18

**Authors:** Monireh Faghir-Gangi, Rozhan Khezri, Yousef Mohammadpour, Rohollah Valizadeh

**Affiliations:** 1 Ph.D. Student in Epidemiology. Email: mfg_1354@yahoo.com, Department of Epidemiology, School of Public Health, Iran University of Medical Sciences, Tehran, Iran. https://orcid.org/0000-0002-4821-5495 Iran University of Medical Sciences Department of Epidemiology School of Public Health Iran University of Medical Sciences Tehran Iran mfg_1354@yahoo.com; 2 Ph.D. Student in Epidemiology. Email: khezri.rojan@yahoo.com. Department of Epidemiology, School of Public Health, Iran University of Medical Sciences, Tehran, Iran. https://orcid.org/0000-0002-7897-9090 Iran University of Medical Sciences Department of Epidemiology School of Public Health Iran University of Medical Sciences Tehran Iran khezri.rojan@yahoo.com; 3 Ph.D. Assistant Professor in Medical Education. Email: mohammadpour.yousef@gmail.com Department of Medical Education, School of Medicine, Urmia University of Medical Sciences, Urmia, Iran https://orcid.org/0000-0001-5078-733X University of Medical Sciences Department of Medical Education School of Medicine Urmia University of Medical Sciences Urmia Iran mohammadpour.yousef@gmail.com; 4 Ph.D. in Epidemiology. Email: rohvali4@gmail.com. Urmia University of Medical Sciences, Urmia, Iran https://orcid.org/0000-0002-6913-3210 Iran University of Medical Sciences Urmia University of Medical Sciences Urmia Iran rohvali4@gmail.com

The COVID-19 pandemic has affected education systems worldwide and led to the closure of face-to-face education in schools and universities. Virtual education has been offered as an alternative to face-to-face teaching in educational centers. Virtual or online education is a type of formal education carried out with the help of electronic resources such as computers and the Internet.^1^ In contrast, face-to-face education, in which the teacher is present in the classroom and communicates verbally with the students simultaneously, is held in fixed physical environments.^2^

## The Challenges of Virtual Education

Before implementing e-learning, providing the required infrastructure and platforms are necessary. In addition, preparing learners and their success in virtual educational environments is critical.^3^ E-learning brings about several challenges for learners, especially in developing countries, including poor internet connection, insufficient knowledge of information and communication technologies, and weakly developed concepts.^4^ Lack of necessary equipment, such as laptops and tablets, due to social or economic issues can cause students to drop out, especially non-disabled students, due to lack of access, lack of equipment, and failure to attend classes. Face-to-face classes can provide a basis for the emergence of mental disorders, including depression and suicide, so it is necessary to provide the essential platforms before implementing electronic or virtual education programs. On the other hand, even if the necessary platforms and facilities are available for providing virtual education, continuous use of tools such as laptops and tablets by students, teachers, professors, and online business owners can create a sedentary lifestyle, which may expose individuals to higher risks for non-communicable diseases. In addition, the long-term use of tools for virtual classes can increase the risk of vision disorders, especially myopia.^5^ Among the other challenges created by e-learning, we can mention the students' shared understanding of course concepts, which causes low motivation for learning. E-learning should be done under specific considerations to avoid cheating and have some practical works involving the applicants. In addition, existing educational weaknesses are not correctly identified due to the lack of face-to-face classes and effective communication between teachers and students.

### Among other challenges that the use of virtual education may create, the following can be mentioned:

Due to the need for financial resources, the lack of sufficient space in homes for studying and teaching, the low level of literacy of parents and students in the digital field, the lack of enough ability to manage cyberspace, the impossibility of monitoring students, the presence of hackers in cyberspace and creating disruptions in these networks, insufficient skills of teachers in providing educational content virtually, the inadequacy of budgeting of lessons with class time, incompatibility of face-to-face education curriculum with virtual education, not giving initiative to teachers in class and continuous monitoring by administrators. Systematic planning in schools and universities, the need to prepare content that can be presented in the form of clips or videos, the lack of sufficient motivation in teachers, and the lack of promotion and payment commensurate with the ability of teachers are challenges and problems for society, student's families, and knowledge.

### Virtual training opportunities

For those interested and motivated to learn, virtual education can give students access to all educational course materials from any location, regardless of their background. Recording the content of virtual training classes and viewing it repeatedly offers students who need more repetition opportunities to use the clips frequently, improving their academic levels in recent years. There is even an opportunity for teachers to pay more attention to their teaching style, take basic measures to improve it, and follow the teaching style of prominent professors. The COVID-19 pandemic has disseminated science and knowledge in a broad geographical scope through virtual education. Many people from different parts of the world could use the content of a virtual education class simultaneously. The COVID-19 pandemic and virtual education caused science publications to rush, even in the post-COVID-19 era. The need to hold virtual classes on different platforms made teachers, students, and students familiar with these platforms and how to use them optimally. In this situation, the students with a strong relationship with technology knew the applications would enjoy them and did not want to return to face-to-face education. The diversity of the learning environment, discretion in choosing the teacher and teaching method, and optimal use of time to learn the material can be attractive and enjoyable for many students and create a positive feeling in them. Saving time and money and not having to travel to and from educational places is another opportunity virtual education can provide. Today, many views on virtual education have changed in the era of COVID-19, and it is unlikely that we will go back to the past. The use of virtual education can reduce traffic and air pollution and thus indirectly reduce DALYs caused by respiratory diseases and driving accidents. Creating employment for some people and guilds, such as programmers, creating virtual scientific associations, and launching national platforms for free virtual teaching and learning in a safe place called home and with the family are other opportunities created by virtual education. And in times of illness, bad weather conditions, and impassable roads, virtual education still provides the opportunity for scientific exploitation. Besides, attention to vaccination in virtual education courses during COVID-19 pandemic is the key issue not only for preventing COVID-19 but also the loss of in-present classes because the main reason for losing classes is fear of severe complications following multi-organ failure and even death resulting in COVID-19 development.^6^^-^^11^

Finally, due to all the limitations, it is impossible to deal with the expansion of technology and modern educational methods, including virtual education. Even with the spread of the COVID-19 vaccine in communities and the presence of schools, universities, and social interactions, virtual education is not terminated. Government officials and administrators have realized the importance of virtual education and consider it an opportunity for the evolution of education. Some measures should be taken so that the main problems of e-learning do not occur, which are poor access to the internet and related hard/software, not having simple educational platforms (not being user-friendly), being affordable to use the internet for lots of hours, involving participants in the discussion, and considering workloads of learner and participants especially in developing countries because it is somehow a new method. In this era, saving time due to not needing to use transportation to attend class, not paying for vehicle, reducing air pollution, and reducing the possibility of contracting COVID-19, is one of the most important benefits of virtual education, and it should also be used in nursing coursework.
